# Clinical Obesity Among Chinese Adults: Prevalence, Multimorbidity Burden, and Associations with Physical Activity

**DOI:** 10.3390/nu18060983

**Published:** 2026-03-19

**Authors:** Zhuoren Wang, Wenwen Du, Xiaoqi Wei, Shujuan Li, Jiguo Zhang, Lahong Ju, Weiyi Gong, Qiya Guo, Xiaoli Xu, Xue Cheng, Ying Xing, Huijun Wang

**Affiliations:** 1 National Institute for Nutrition and Health, Chinese Center for Disease Control and Prevention, Beijing 100050, China; wangzhuoren2012@126.com (Z.W.);; 2 Key Laboratory of Public Nutrition and Health, National Health Commission of the People’s Republic of China, Beijing 100050, China

**Keywords:** clinical obesity, preclinical obesity, excess adiposity, multimorbidity, prevalence, China

## Abstract

**Background/Objectives:** The Lancet Diabetes & Endocrinology Commission recently proposed distinguishing preclinical obesity from clinical obesity, in which excess adiposity is accompanied by obesity-related disease. Using data from the China Nutrition and Health Surveillance 2015, we estimated the prevalence and comorbidity burden of clinical obesity among Chinese adults and further explored the association between physical activity and clinical obesity. **Methods:** This cross-sectional study included adults aged 18–79 years from the China Nutrition and Health Surveillance 2015 (*N* = 180,480). Excess adiposity was identified using body mass index, waist circumference, and waist-to-height ratio. Participants were classified as having no obesity, preclinical obesity, or clinical obesity, defined as excess adiposity plus at least one obesity-related comorbidity. We estimated the prevalence of clinical obesity overall and across sociodemographic subgroups. In secondary analyses, we examined the association of physical activity (MET-min/week) with clinical obesity prevalence and ordered obesity status using fully adjusted proportional-odds models. **Results:** Overall, 33.9% of adults had excess adiposity, and 26.9% met the criteria for clinical obesity. The prevalence of clinical obesity increased from 15.3% among adults aged 18–39 years to 33.3% among those aged ≥60 years and varied across sociodemographic subgroups. Among individuals with clinical obesity, dyslipidemia, hypertension, and diabetes were the most common comorbidities, indicating a substantial cardiometabolic burden. Higher physical activity was associated with a lower prevalence of clinical obesity and lower odds of more severe ordered obesity status. **Conclusions:** Clinical obesity affects more than one quarter of Chinese adults and is associated with a substantial cardiometabolic burden. Higher physical activity was associated with a lower prevalence of clinical obesity and less severe obesity status. These findings provide nationally relevant evidence to inform prevention and clinical management in China.

## 1. Introduction

Obesity is a major public health concern worldwide and in China and is associated with substantial cardiometabolic and other chronic disease burdens. In China, the number of adults affected by obesity-related health consequences continues to increase, with pronounced variation across regions and sociodemographic groups, posing challenges for prevention and health-system planning [1]. Recent evidence indicates that both general obesity and abdominal obesity have increased substantially in China over recent decades, underscoring the need for surveillance approaches that better capture clinically meaningful health burden rather than body size alone [2].

To better align obesity assessment with clinically meaningful outcomes, the recent Lancet Diabetes & Endocrinology Commission and accompanying editorial proposed redefining obesity as a disease state and introduced 2 related constructs: preclinical obesity (excess adiposity without obesity-attributable organ dysfunction or functional impairment) and clinical obesity (excess adiposity with obesity-related organ dysfunction or functional impairment) [3,4]. The Commission further recommended integrating adiposity indicators beyond BMI (e.g., central adiposity measures) with clinical manifestations to support population monitoring and clinical decision-making [3]. This recommendation is particularly relevant in Asian populations, because BMI does not distinguish fat mass from lean mass and may not adequately capture excess central adiposity or related cardiometabolic risk at relatively lower BMI levels [5]. In Chinese adults, anthropometric indicators such as waist circumference (WC) and waist-to-height ratio (WHtR) may therefore provide additional value in identifying adiposity-related cardiometabolic risk [6].

Early applications of the commission framework suggest that prevalence estimates and subgroup patterns can shift meaningfully when “clinical obesity” criteria are applied. Analyses using NHANES and other US data have quantified clinical obesity prevalence under the proposed definitions, and related cohort work indicates that clinical classifications may identify risk profiles that are not fully captured by BMI-defined obesity, with similar implications evaluated in large-scale resources such as All of Us [7,8,9,10]. Emerging longitudinal evidence further supports the prognostic relevance of distinguishing preclinical from clinical obesity: in the UK Biobank, clinical obesity (vs non-obesity/preclinical obesity) has been associated with higher all-cause mortality, and clinical obesity phenotypes have also been linked to elevated incidence risks for selected chronic conditions [11,12]. In addition, pooled analyses across national health surveys indicate that adopting clinical criteria can materially revise population prevalence estimates and shift attention toward metabolic/functional impairment beyond BMI [13].

At the same time, research on factors associated with clinical obesity remains very limited, especially in nationally representative populations. Although previous studies have shown that physical activity (PA) is an important modifiable factor in obesity prevention and chronic disease control and that higher PA levels are generally associated with lower risks of general obesity, central obesity, and adverse metabolic outcomes, evidence specifically examining its role in clinically oriented obesity stages is still insufficient [14,15]. Further evidence is therefore needed to clarify the relationship between PA and clinical obesity within this newly proposed framework.

Despite rapidly expanding evidence from Western cohorts, nationally representative estimates of preclinical and clinical obesity in China remain limited. This gap matters because body composition, central adiposity distribution, and cardiometabolic risk at a given BMI may differ between Asian and Western populations, and because policy planning requires estimates that more directly reflect healthcare burden and intervention priorities [1,3]. Applying a clinically oriented obesity framework in China may therefore improve the identification of high-burden subgroups and provide more actionable evidence for chronic disease prevention, public health intervention planning, and healthcare resource allocation. Because CNHS is a surveillance dataset with more complete ascertainment of cardiometabolic conditions than of functional impairment or multisystem organ dysfunction, the present classification should be interpreted as a surveillance-based operationalization of the Lancet framework. Therefore, using data from the China Nutrition and Health Surveillance (CNHS)—a large, nationally representative cross-sectional survey with standardized questionnaires, anthropometric assessments, and health measurements [16]—we aimed to: (1) estimate the prevalence of preclinical and clinical obesity among Chinese adults; (2) describe differences by sex, age, residence, and region; (3) characterize the comorbidity burden among adults with clinical obesity; and (4) examine the association between physical activity level and ordered obesity status in the overall population and key subgroups.

## 2. Materials and Methods

**Figure 1:**
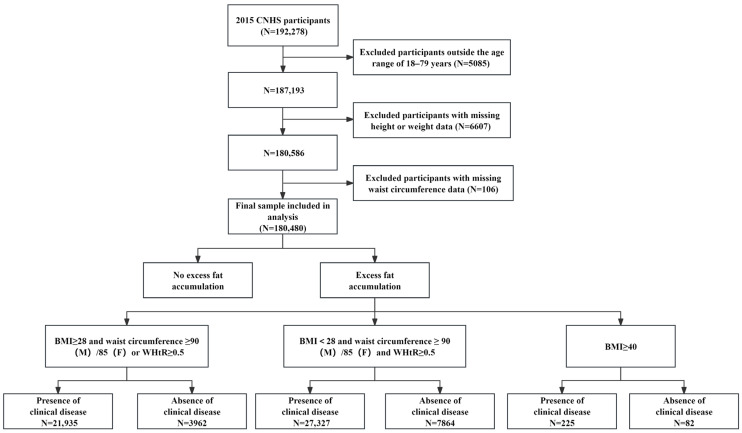
Study flowchart and operational classification algorithm. ***Notes:*** Participants were from CNHS 2015 and restricted to adults aged 18–79 years. Individuals were excluded if their age was out of range or if their height/weight or waist circumference data were missing. “Excess adiposity” was operationalized using BMI and central adiposity indicators: (1) BMI ≥ 28 kg/m^2^ and (WC ≥ 90 cm in men or ≥85 cm in women or WHtR ≥ 0.50); (2) BMI < 28 kg/m^2^ and (WC ≥ 90 cm in men or ≥85 cm in women and WHtR ≥ 0.50); or (3) BMI ≥ 40 kg/m^2^. Among participants with excess adiposity, “presence of clinical disease” indicates clinical obesity, whereas “absence of clinical disease” indicates preclinical obesity (clinical diseases defined as in Methods). Numbers shown in the flowchart are unweighted counts (*n*).

We conducted a cross-sectional analysis of the CNHS 2015 to operationalize a clinically oriented obesity classification and to estimate prevalence, comorbidity burden, and associations with physical activity. This study flowchart and operational classification algorithm (including participant selection and obesity-status construction) are presented in Figure 1.

### 2.1. Study Design and Participants

CNHS 2015 is a cross-sectional survey conducted across 31 provincial-level administrative divisions in China. Participant selection is shown in Figure 1. Among 192,278 participants, we excluded those outside the eligible age range of 18–79 years (*n* = 5085), those with missing height or weight (*n* = 6607), and those with missing waist circumference (*n* = 106). The final analytic sample included 180,480 adults (84,065 men and 96,415 women). To improve national representativeness, all analyses incorporated survey population weights, stratification, and clustering variables, and the estimates were weighted to the national adult population based on data from the Sixth National Population Census.

### 2.2. Measurements and Definitions

Anthropometric measurements were collected by trained staff using standardized protocols. Height was measured using a TZG stadiometer (Wuxi Weighing Apparatus Factory Co., Ltd., Wuxi, China) to the nearest 0.1 cm, and weight was measured using a TANITA HD-390 electronic scale (Dongguan Tanita Health Equipment Co., Ltd., Dongguan, China) to the nearest 0.1 kg. Waist circumference (WC) was measured with a standardized waist tape to the nearest 0.1 cm. Body mass index (BMI) was calculated as weight (kg) divided by height squared (m^2^), and waist-to-height ratio (WHtR) was computed as WC (cm)/height (cm). Blood pressure was measured using an Omron HBP-1300 electronic sphygmomanometer (Omron (Dalian) Co., Ltd., Dalian, China) (measurement range 0–300 mmHg), with blood pressure recorded to the nearest 1 mmHg.

Physical activity (PA) was assessed by questionnaire and summarized as MET-min/week. Following the equivalence principle that 1 min of vigorous-intensity activity equals 2 min of moderate-intensity activity, we converted all reported activity duration into moderate-intensity equivalent minutes. MET values were assigned as 4 for moderate-intensity equivalent activity, and total PA volume was expressed as MET-min/week. PA categories were defined to create an ordered gradient of weekly activity with reasonably balanced group sizes across the study population while retaining a clinically interpretable threshold at 600 MET-min/week, which corresponds to the WHO minimum recommendation. Specifically, <600 MET-min/week represented not meeting the WHO minimum recommendation, 600–1200 MET-min/week represented meeting the minimum up to approximately twice the minimum, and higher cut points captured progressively greater volumes (1200–3000 and ≥3000 MET-min/week). We additionally split the sub-threshold range at 300 MET-min/week to distinguish near-inactive participants from those engaging in low but nonzero activity.

Excess adiposity was defined using an anthropometric algorithm incorporating BMI and central adiposity indicators. To better capture adiposity-related health risk, this algorithm combined BMI with central adiposity indicators rather than relying on BMI alone. Participants were classified as having excess adiposity if they met any of the following criteria: (1) BMI ≥ 28.0 kg/m^2^ and (high WC or WHtR ≥ 0.50); (2) BMI < 28.0 kg/m^2^ and (high WC and WHtR ≥ 0.50); or (3) BMI ≥ 40.0 kg/m^2^. High WC was defined as ≥90 cm in men and ≥85 cm in women. These cut-offs were selected to align with commonly used Chinese adult thresholds for obesity and abdominal obesity and with the widely used WHtR threshold of 0.50. Among participants with excess adiposity, obesity status was staged based on the presence of obesity-related clinical disease: preclinical obesity (excess adiposity without obesity-related clinical disease) and clinical obesity (excess adiposity with obesity-related clinical disease).

Obesity-related clinical diseases included dyslipidemia, hypertension (HTN), diabetes mellitus (DM), chronic obstructive pulmonary disease (COPD), ischemic stroke, asthma, angina, malignancy, myocardial infarction (MI), atrial fibrillation (AF), hemorrhagic stroke, percutaneous coronary intervention/stent (PCI/stent), and coronary artery bypass grafting (CABG). Dyslipidemia was defined as any of the following: total cholesterol ≥ 6.2 mmol/L, triglycerides ≥ 2.3 mmol/L, LDL-C ≥ 4.1 mmol/L, or HDL-C < 1.0 mmol/L, and/or self-reported physician diagnosis and/or lipid-lowering medication use. HTN was defined as measured SBP ≥ 140 mmHg and/or DBP ≥ 90 mmHg, and/or self-reported physician diagnosis, and/or antihypertensive medication use. DM was defined as fasting plasma glucose ≥ 7.0 mmol/L, 2 h OGTT glucose ≥ 11.1 mmol/L, HbA1c ≥ 6.5%, or random glucose ≥ 11.1 mmol/L with typical symptoms, and/or self-reported physician diagnosis, and/or glucose-lowering medication use. The remaining conditions (COPD, ischemic stroke, asthma, angina, malignancy, MI, AF, hemorrhagic stroke, PCI/stent, and CABG) were ascertained via questionnaire-based self-reported physician diagnosis. Only a small proportion of individuals classified as having clinical obesity (approximately 11%) were identified on the basis of self-reported conditions alone.

### 2.3. Statistical Analysis

Categorical variables are presented as numbers (percentages), and continuous variables as mean (standard deviation). All descriptive and regression analyses were conducted using survey weights, stratification, and clustering variables to account for the complex sampling design and to obtain nationally representative estimates. Prevalence estimates are reported with 95% confidence intervals (CIs) based on binomial methods. Subgroup comparisons used χ^2^ tests for categorical variables and *t* tests/ANOVA for continuous variables, as appropriate.

We estimated the prevalence of clinical obesity overall and by sex, age group, residence, region, and PA level. Among adults with clinical obesity, we described comorbidity profiles and comorbidity counts by age group. To evaluate associations between PA level and ordered obesity status, we fitted proportional odds ordinal logistic regression models and reported cumulative odds ratios (ORs) with 95% CIs. The proportional odds assumption was tested before model interpretation and was considered acceptable for the final models. Sequential models were specified a priori: Model 1 (crude); Model 2 (age group); Model 3 (add residence and region); and Model 4 (add smoking, alcohol drinking, total energy intake, and percent energy from fat). Analyses were conducted overall and stratified by sex. Model-specific sample sizes reflect complete-case availability of covariates, and Model 4 was restricted to participants with complete dietary and lifestyle covariate data. Accordingly, the fully adjusted estimates should be interpreted cautiously in light of potential selection bias related to covariate missingness. Subgroup analyses were interpreted as exploratory, and no formal correction for multiple testing was applied. All analyses were performed in R version 4.4.3 (R Foundation for Statistical Computing, Vienna, Austria).

### 2.4. Ethics

This study protocol was evaluated by the Ethics Committee of the Chinese Center for Disease Control and Prevention (China CDC) (No. 201519-B, date: 2015.06.15). All information was collected by trained investigators, and the participants in this survey voluntarily participated and signed an informed consent form.

## 3. Results

First, we describe the study population and participant characteristics. Then, we report the prevalence of preclinical and clinical obesity overall and across key subgroups. Finally, we summarize comorbidity patterns among adults with clinical obesity and examine associations between physical activity and ordered obesity status.

### 3.1. Study Population and Participant Characteristics

A total of 180,480 adults were included in the analysis. Table 1 shows the basic characteristics of the study population. Nearly half were aged 40–59 years (47.8%), and 30.9% were aged ≥ 60 years; 59.4% resided in rural areas, with a broadly even geographic distribution across eastern, central, and western regions. The mean BMI was 24.3 kg/m^2^. Using Chinese BMI cut-points, 35.2% of participants were classified as overweight, and 14.6% were classified as having obesity. Central adiposity was also common. Based on waist-circumference thresholds (men ≥ 90 cm; women ≥ 85 cm), 32.8% of participants exceeded the cut-off. Physical activity showed substantial heterogeneity. The overall mean physical activity was 1537 MET-min/week (Table 1).

### 3.2. Distribution of Excess Adiposity Classifications

Overall, 33.9% of adults met the criteria for excess adiposity under the Commission-aligned classification. The most common phenotype was central adiposity with BMI < 28.0 kg/m^2^, defined by elevated waist circumference (men ≥ 90 cm/women ≥ 85 cm) and WHtR ≥ 0.50, affecting 19.5% of adults. BMI-defined obesity with additional central adiposity indicators (BMI ≥ 28.0 kg/m^2^ plus either elevated waist circumference or WHtR ≥ 0.50) accounted for 14.3%.

Women had a higher prevalence of excess adiposity than men (35.8% vs. 31.6%), primarily driven by a higher prevalence of central adiposity with BMI < 28.0 kg/m^2^ (20.8% vs. 18.0%). The prevalence of BMI ≥ 28.0 kg/m^2^ with central adiposity indicators was similar between women and men (14.9% vs. 13.6%) (Table 2).

### 3.3. Prevalence of Preclinical and Clinical Obesity

**Figure 2:**
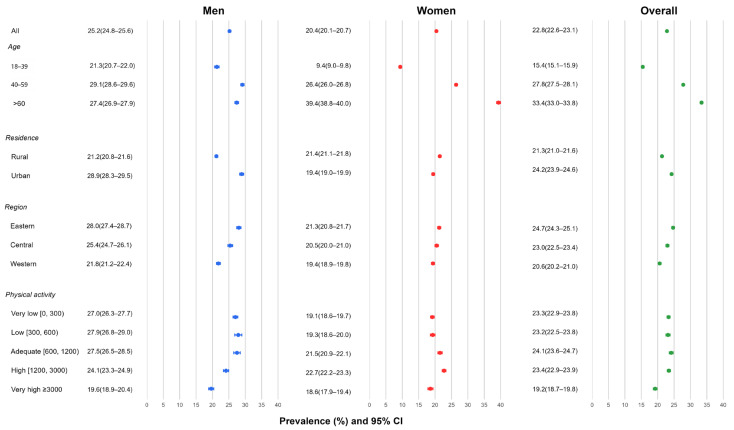
Prevalence of clinical obesity by sex and population subgroups in CNHS 2015. ***Notes:*** Blue indicates men, red indicates women, and green indicates overall estimates. Points indicate prevalence estimates, and horizontal lines represent 95% confidence intervals. Estimates are shown overall and stratified by age group (18–39, 40–59, ≥60 years), residence (rural/urban), region (Eastern/Central/Western), and physical activity category (MET-min/week: very low 0–300, low 300–600, medium 600–1200, high 1200–3000, very high ≥3000). Clinical obesity was defined as excess adiposity with ≥1 obesity-related clinical disease.

Among 180,480 adults included in the CNHS 2015 analytic sample, 33.9% met the criteria for excess adiposity. The overall prevalence of clinical obesity was 26.9% (95% CI 26.7–27.1), with a slightly higher prevalence in women than men (27.4% vs. 26.4%). The prevalence of preclinical obesity was 7.0% overall (8.4% in women and 5.2% in men). Among adults with excess adiposity, a larger proportion were classified as clinically obese in men than in women (approximately 83.5% vs. 76.5%) (Figure 2).

Clinical obesity prevalence varied across subgroups and differed by sex. By age, prevalence was 15.3% in adults aged 18–39 years, 27.9% in those aged 40–59 years, and 33.3% in those aged ≥ 60 years overall. Sex differences showed an age-by-sex crossover: women had a markedly lower prevalence than men in the 18–39-year group, estimates were similar in the 40–59-year group, and women had a higher prevalence than men in the ≥60-year group. In the 18–39-year group, prevalence was higher in men than women (21.6% vs. 10.2%); in the ≥60-year group, prevalence was higher in women than men (39.4% vs. 27.1%), while estimates were similar in the 40–59-year group (28.0% in men vs. 27.9% in women). By residence, prevalence was higher in urban than rural participants overall (30.3% vs. 24.6%); however, sex patterns differed by residence, with women exceeding men in rural areas (26.7% vs. 22.3%) and men exceeding women in urban areas (32.7% vs. 28.4%). Regionally, prevalence was highest in Eastern China (29.8%) and lowest in Western China (24.1%). Across physical activity categories, prevalence was highest in the very low group (0–300 MET-min/week; 28.6%) and lowest in the very high group (≥3000 MET-min/week; 20.6%). Men had higher prevalence than women in the very low (29.5% vs. 27.7%) and low activity groups (30.3% vs. 27.4%), while women had higher prevalence than men in the high (28.5% vs. 24.7%) and very high activity groups (22.3% vs. 19.3%) (Figure 2).

### 3.4. Comorbidity Burden and Profiles Among Adults with Clinical Obesity

**Figure 3:**
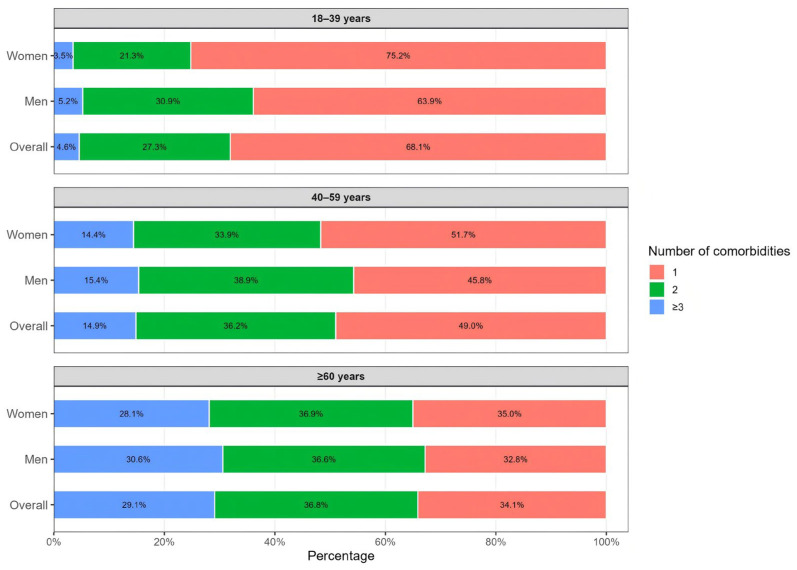
Distribution of comorbidity counts among adults with clinical obesity in CNHS 2015. ***Notes:*** Stacked bars show the percentage of adults with clinical obesity who had 1, 2, or ≥3 obesity-related comorbidities, presented overall and by sex within each age group (18–39, 40–59, and ≥60 years). Percentages may not sum to 100% due to rounding.

Among adults with clinical obesity, multimorbidity was common and increased markedly with age, with a clear shift in the distribution of comorbidity counts from younger to older groups. In participants aged 18–39 years, the burden was predominantly low, as 68.1% had one comorbidity, 27.3% had two, and 4.6% had ≥3. In the 40–59 years group, the distribution moved toward higher burden, with the share with one comorbidity decreasing to 49.0% and the share with ≥3 comorbidities increasing to 14.9%, while 36.2% had two comorbidities. Among adults aged ≥60 years, the burden increased further: 34.1% had one comorbidity, 36.8% had two (the most common pattern), and 29.1% had ≥3. Overall, the proportion with ≥3 comorbidities rose steadily across age groups, from 4.6% in 18–39 years to 14.9% in 40–59 years and 29.1% in ≥60 years, while the proportion with only one comorbidity declined in parallel from 68.1% to 49.0% and then to 34.1%. Sex patterns were broadly similar within each age stratum; however, in the ≥60 years group, men had a slightly higher proportion with ≥3 comorbidities than women, at 30.6% versus 28.1% (Figure 3).

**Figure 4:**
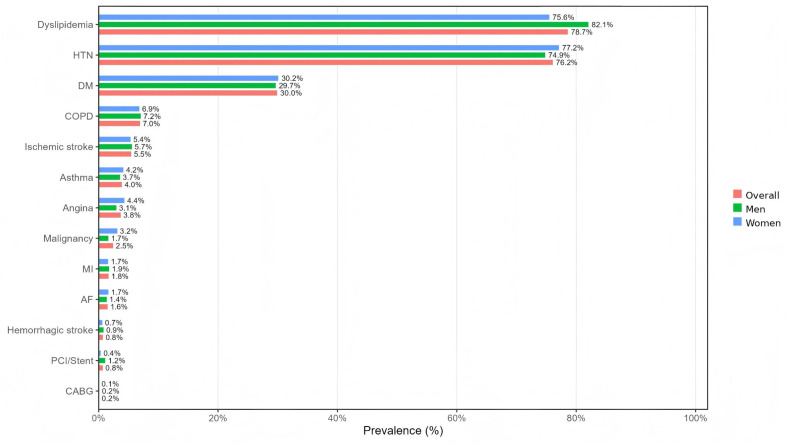
Prevalence of specific comorbidities among adults with clinical obesity. ***Notes:*** Bars show the prevalence (%) of each comorbidity among adults with clinical obesity, presented overall and by sex. Comorbidities include dyslipidemia, hypertension (HTN), diabetes mellitus (DM), chronic obstructive pulmonary disease (COPD), ischemic stroke, hemorrhagic stroke, asthma, angina, malignancy, myocardial infarction (MI), atrial fibrillation (AF), percutaneous coronary intervention/stent (PCI/stent), and coronary artery bypass grafting (CABG). Clinical obesity was defined as excess adiposity with ≥1 obesity-related clinical disease.

Cardiometabolic conditions accounted for the dominant comorbidity profile among adults with clinical obesity (Figure 4). Dyslipidemia and hypertension were the two most prevalent conditions overall, followed by diabetes mellitus and chronic obstructive pulmonary disease. The composition differed modestly by sex: dyslipidemia was more common in men than women (82.1% vs. 75.6%), whereas hypertension was slightly higher in women than men (77.2% vs. 74.9); diabetes and COPD showed minimal sex differences. These cardiometabolic conditions should be interpreted as major defining components of the present operational classification of clinical obesity rather than fully independent downstream outcomes (Figure 4).

### 3.5. Association Between Physical Activity and Ordered Obesity Status

**Figure 5:**
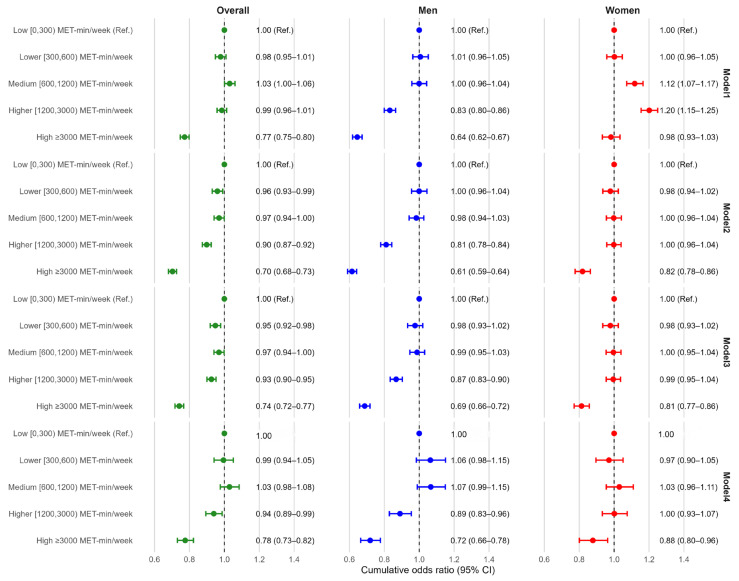
Association between physical activity and ordered obesity status. ***Notes:*** The very low PA group served as the reference category. Green indicates overall estimates, blue indicates men, and red indicates women. Points show cumulative odds ratios (cORs), and horizontal lines show 95% confidence intervals from proportional odds ordinal logistic regression models for higher ordered obesity status (no obesity → preclinical obesity → clinical obesity), presented overall and by sex. Physical activity (PA) was categorized by MET-min/week as very low (0–300), low (300–600), medium (600–1200), high (1200–3000), and very high (≥3000). The proportional odds assumption was tested before model interpretation and was considered acceptable for the final models. Model 1: unadjusted (crude). Model 2: adjusted for age group. Model 3: additionally adjusted for residence (rural/urban) and region (Eastern/Central/Western). Model 4: additionally adjusted for smoking, alcohol drinking, total energy intake, and percent energy from fat (restricted to participants with complete dietary and lifestyle covariate data). Models 1–3 used *n* = 177,411, and Model 4 used *n* = 60,322.

In ordinal logistic regression models assessing the association between physical activity (PA) and higher-order obesity status, a clear inverse gradient was observed at higher PA levels, particularly among men. Models 1–3 were estimated in participants with complete data on obesity status, PA, age, residence, and region (*N* = 177,411). Model 4 additionally adjusted for smoking, alcohol drinking, total energy intake, and percent energy from fat and was therefore restricted to participants with complete dietary and lifestyle covariate data (*N* = 60,322). Using the very low PA group (0–300 MET-min/week) as the reference, the fully adjusted model (Model 4) showed that high PA (1200–3000 MET-min/week) was associated with lower cumulative odds of a higher obesity category (cOR 0.88, 95% CI 0.84–0.93), and very high PA (≥3000 MET-min/week) showed a stronger inverse association (cOR 0.70, 95% CI 0.66–0.74). In contrast, low PA (300–600 MET-min/week) and medium PA (600–1200 MET-min/week) were not materially different from the reference group (Figure 5).

## 4. Discussion

### 4.1. Study Strengths

This study used nationally representative CNHS 2015 data covering 31 provincial-level administrative divisions, providing precise population estimates and enabling prespecified stratified analyses by sex, age, residence, and region. We moved beyond BMI-only classification by integrating BMI with central adiposity measures (waist circumference and waist-to-height ratio using China-specific cut points) to define excess adiposity and by staging obesity according to obesity-related clinical disease, thereby improving clinical relevance. Standardized anthropometry, measured blood pressure, and laboratory cardiometabolic markers strengthened outcome ascertainment, and ordinal logistic regression with sequential covariate adjustment supported the robustness and interpretability of associations between physical activity and obesity severity.

### 4.2. The Distribution of Excess Adiposity

In CNHS 2015, BMI-based categories suggested that nearly half of adults were classified as having overweight or obesity (49.8%). However, when excess adiposity was operationalized using a Commission-aligned anthropometric algorithm that incorporated central adiposity, only one-third of adults met criteria (33.9%). Importantly, the dominant excess-adiposity phenotype was central adiposity with BMI below the Chinese obesity threshold (BMI < 28.0 kg/m^2^ plus elevated waist circumference and WHtR ≥ 0.50), affecting 19.5% of adults and accounting for 57.3% of all excess adiposity cases. These findings underscore that BMI-only surveillance can miss a large subgroup with clinically relevant central fat accumulation at subthreshold BMI while also grouping together heterogeneous risk profiles within BMI-defined “overweight/obesity”.

This pattern is particularly salient for Asian populations, where cardiometabolic risk can occur at lower BMI levels, and population-specific interpretation of BMI cut points has been emphasized by the WHO Expert Consultation [5]. Consistent with this, international evidence suggests that abdominal adiposity metrics improve risk stratification: in the INTERHEART case–control study across 52 countries, the waist-to-hip ratio showed a strong graded association with myocardial infarction risk and yielded substantially higher population-attributable risk estimates than BMI [17]. In China, national surveillance analyses similarly indicate that central obesity remains common (standardized prevalence 29.9%), supporting the need to integrate waist-based indicators into population phenotyping rather than relying on BMI alone [15].

Our findings also align with large US cohorts demonstrating substantial reclassification, from 42.9% under traditional BMI-based criteria to 68.6% under a new definition that integrated BMI with anthropometric measures; this increase was driven by “anthropometric-only obesity”, with 25.9% of participants reclassified as having obesity despite not meeting the traditional BMI threshold [10]. In NHANES 2017–2018, BMI-based obesity (43.81%) and clinical obesity (44.74%) were similar in overall prevalence, yet overlap between definitions was limited (25.76% met both), indicating meaningful reclassification when clinical criteria were applied [7]. Although cross-country comparisons are not one-to-one given different BMI cut points and operational measures, together these data support a multi-metric approach to identifying excess adiposity and related risk—especially in populations where central adiposity at lower BMI is common.

### 4.3. Prevalence of Clinical Obesity

In CNHS 2015, approximately one-third of adults met criteria for excess adiposity, and most of this burden clustered in clinical obesity rather than preclinical obesity (clinical obesity, 26.9%; preclinical, 7.0%). Clinical obesity increased stepwise with age (18–39 years, 15.3%; 40–59 years, 27.9%; ≥60 years, 33.3%). This age gradient is directionally consistent with estimates from other large population-based samples applying the Lancet Commission framework, including NHANES (clinical obesity prevalence increasing sharply with age) and KNHANES (clinical obesity 31.2%; preclinical obesity 8.1%) [7,18].

A marked age-by-sex crossover emerged. Women had substantially lower clinical obesity prevalence than men in early adulthood (18–39 years: 10.2% vs. 21.6%), little difference in midlife (40–59 years: 27.9% vs. 28.0%), and higher prevalence after age 60 years (39.4% vs. 27.1%). Notably, although women had higher excess adiposity overall (driven by central adiposity at BMI < 28.0), men with excess adiposity were more often classified as clinical rather than preclinical obesity (≈83.5% vs. 76.5%), suggesting earlier accumulation of obesity-related dysfunction in men. This pattern aligns with evidence that cardiometabolic risk profiles differ by sex and evolve across adulthood, and it is biologically plausible that the postmenopausal transition contributes to later-life increases in central adiposity and cardiometabolic vulnerability among women [19,20].

This sex crossover is also compatible with China-wide evidence that adiposity phenotypes differ by sex across the life course: in a large national analysis of abdominal obesity, the prevalence of overweight/obesity was higher in men before approximately age 45 years but higher in women thereafter, and abdominal obesity is often more prevalent in women [21,22]. Taken together, these data suggest that (1) men may transition to clinical obesity earlier because obesity-related cardiometabolic abnormalities emerge sooner, whereas (2) women may carry central adiposity for longer without meeting clinical criteria until midlife-to-late-life, when hormonal and metabolic changes increase the likelihood of obesity-related dysfunction [19,20].

From a public health perspective, these patterns suggest that a clinically oriented obesity framework may help identify subgroups with a higher burden of obesity-related dysfunction more effectively than BMI-based classification alone, thereby informing targeted prevention, screening, and healthcare planning in China.

### 4.4. Multimorbidity Profiles and Aging

In CNHS 2015, the comorbidity profile of clinical obesity was dominated by cardiometabolic conditions, with dyslipidemia and hypertension as the most prevalent conditions, followed by diabetes; respiratory disease (e.g., COPD), cerebrovascular disease (e.g., ischemic/hemorrhagic stroke), coronary disease manifestations (e.g., angina, myocardial infarction, revascularization), atrial fibrillation, and malignancy were also captured, underscoring that clinical obesity reflects a multisystem burden rather than excess weight alone. This pattern is conceptually concordant with the Lancet Commission’s framing of clinical obesity as excess adiposity with organ dysfunction and/or functional impairment, rather than a pure anthropometric label [3].

A pronounced age gradient was observed in CNHS: the distribution of comorbidity counts shifted steadily toward higher multimorbidity with aging (e.g., the proportion with ≥3 comorbidities increased from younger to older groups). Similar age patterns have been reported in large, nationally representative US data—NHANES showed that clinical obesity prevalence rose sharply with age (reaching very high levels in the oldest strata), even when BMI-based obesity did not increase in parallel [7]. In Korea, application of the same clinical framework to the KNHANES likewise suggested that clinical obesity increased with age despite stable BMI, consistent with cumulative metabolic and functional decline over the life course [18].

Across settings, cardiometabolic dysfunction appears to be the most consistently operationalized component of clinical obesity in population data. In NHANES, the operational criteria for clinical obesity included elevated blood pressure and a metabolic cluster incorporating glycemia and lipids, alongside kidney function (eGFR) and NAFLD-related measures, plus other systems when available [7]. In a pooled analysis across 56 national health surveys, “clinical obesity” was pragmatically implemented using cardiometabolic complications (e.g., diabetes, hypertension, total cholesterol) in combination with anthropometric thresholds, reinforcing that metabolic disease is often the most feasible cross-country anchor for surveillance definitions [13].

Between-country differences likely reflect (1) which organ dysfunctions are measurable, (2) how comprehensively functional limitation is captured, and (3) underlying population disease epidemiology. NHANES operationalization explicitly included sleep apnea, urinary incontinence, musculoskeletal limitations, and activity limitations when available, in addition to cardiometabolic and hepatorenal dysfunction [7]. By contrast, in many surveillance systems (including China’s large-scale monitoring), functional limitation and some organ-specific assessments may be less completely measured, which can shift the observed “burden mix” toward cardiometabolic diagnoses. In addition, large US cohorts show that clinical obesity status strongly stratifies downstream outcomes: in All of Us, clinical obesity was associated with markedly higher risk of incident diabetes, cardiovascular events, and mortality during follow-up, supporting the interpretation that clinical obesity captures an advanced burden phenotype; this provides an external benchmark when discussing why older adults disproportionately concentrate multimorbidity [10].

Taken together, similarities across CNHS, NHANES, and KNHANES suggest that cardiometabolic disease is the most stable cross-setting signature of clinical obesity, whereas differences in comorbidity composition likely arise from heterogeneous implementation of the clinical criteria (availability of functional limitation measures and organ-specific tests), differences in age structure and case detection, and population variation in adiposity distribution and severity. Prospective evidence from the UK Biobank further indicates that clinical obesity confers the highest long-term mortality risk (with preclinical obesity also carrying elevated risk), emphasizing that multimorbidity and aging patterns observed in CNHS plausibly represent clinically meaningful risk stratification rather than surveillance artifact [12,23].

These findings further indicate that clinically oriented obesity classification may be useful not only for prevalence estimation but also for recognizing groups with concentrated multimorbidity burden, particularly older adults who may require more intensive chronic disease management and integrated care.

### 4.5. Physical Activity and Ordered Obesity Status

In our CNHS analysis, clinical obesity prevalence decreased across physical activity (PA) categories, from 28.6% in the very-low PA group (0–300 MET-min/week) to 20.6% in the very-high PA group (≥3000 MET-min/week). In the fully adjusted model, high and very high PA were associated with lower cumulative odds of more severe ordered obesity status (no obesity → preclinical obesity → clinical obesity) (cOR, 0.88 and 0.70, respectively). These findings suggest an inverse association between higher PA and obesity severity in this nationally representative sample.

Consistent with our findings, longitudinal data from the UK Biobank suggest that increasing physical activity to guideline levels is associated with a lower risk of incident obesity [24]. Among 31,344 adults without obesity at baseline followed for a mean of 6.8 years, participants who moved from insufficient activity to meeting physical activity guidelines at follow-up had lower odds of developing obesity (OR, 0.71; 95% CI, 0.58–0.85), similar to those who met guidelines at both time points (OR, 0.71; 95% CI, 0.58–0.87) [24]. Evidence from China Nutrition and Health Surveillance (2015–2017; *n* = 145 298) further supports this pattern, with sufficient physical activity associated with lower odds of central obesity (OR, 0.819; 95% CI, 0.782–0.858) [15]. Overall, these findings align with WHO public health recommendations emphasizing the health benefits of higher levels of physical activity [25].

However, these associations should be interpreted cautiously. Because the present analysis is cross-sectional, causality cannot be inferred, and reverse causation is possible. Individuals with more severe obesity-related disease burden may reduce their physical activity because of existing symptoms, functional limitations, or medical advice, which could partly contribute to the observed inverse association. In addition, PA in CNHS was assessed using questionnaire-based interviewer-administered data, which may still be affected by recall bias, social desirability bias, and measurement error. Therefore, although our findings support an association between higher PA and lower obesity severity, prospective studies using repeated or objective PA assessment are needed to clarify temporality and causality.

### 4.6. Study Limitations

Several limitations should also be acknowledged. First, because CNHS is a surveillance dataset in which disease ascertainment is more heavily oriented toward cardiometabolic conditions, while complete measures of functional impairment and multisystem organ dysfunction were not available, the present classification should be interpreted as a surveillance-based operationalization of the Lancet framework rather than a full implementation of the Commission definition. Second, among individuals classified as having clinical obesity, only a small proportion (approximately 11%) were identified on the basis of self-reported conditions alone; therefore, some degree of misclassification cannot be excluded. Third, Model 4 was restricted to participants with complete dietary and lifestyle covariate data, and therefore, potential selection bias cannot be excluded. Finally, because the present analysis was observational and cross-sectional, residual confounding and reverse causation cannot be excluded, and causal inference is limited.

## 5. Conclusions

In this nationally representative sample of Chinese adults, excess adiposity was common, and most affected individuals were classified as having clinical rather than preclinical obesity. Clinical obesity showed clear variation by sex and age, and its comorbidity profile was dominated by cardiometabolic conditions, with multimorbidity becoming more pronounced in older adults. Higher physical activity levels were associated with lower odds of more severe ordered obesity status. These findings suggest that a clinically oriented obesity framework may provide more meaningful information than BMI-based classification alone for identifying high-burden subgroups and informing chronic disease prevention and healthcare planning in China. However, because the present analysis was cross-sectional and partly relied on self-reported data, prospective studies are needed to confirm temporality and to further evaluate the utility of clinically oriented obesity staging in Chinese populations.

## Figures and Tables

**Table 1:** Participant characteristics of the study population (CNHS 2015).

Variable	Overall	Men	Women	*p*
Sample size (*N*)	180,480	84,065	96,415	
Age group (years)				<0.001
18–39	38,387 (21.3%)	17,143 (20.4%)	21,244 (22.0%)	
40–59	86,289 (47.8%)	39,302 (46.8%)	46,987 (48.7%)	
≥60	55,804 (30.9%)	27,620 (32.9%)	28,184 (29.2%)	
Residence				<0.001
Rural	105,449 (59.4%)	50,274 (60.8%)	55,175 (58.1%)	
Urban	72,218 (40.6%)	32,443 (39.2%)	39,775 (41.9%)	
Region				0.239
Eastern	65,875 (36.5%)	30,516 (36.3%)	35,359 (36.7%)	
Central	50,879 (28.2%)	23,739 (28.2%)	27,140 (28.1%)	
Western	63,726 (35.3%)	29,810 (35.5%)	33,916 (35.2%)	
Body mass index (BMI), kg/m^2^, mean (SD)	24.3 (3.8)	24.2 (3.8)	24.3 (3.8)	<0.001
BMI categories (kg/m^2^)				<0.001
<18.5	6488 (3.6%)	2814 (3.3%)	3674 (3.8%)	
18.5 ≤ BMI < 24.0	84,132 (46.6%)	39,556 (47.1%)	44,576 (46.2%)	
24.0 ≤ BMI < 28.0	63,478 (35.2%)	30,043 (35.7%)	33,435 (34.7%)	
≥28.0	26,382 (14.6%)	11,652 (13.9%)	14,730 (15.3%)	
Waist-to-height ratio (WHtR), mean (SD)	0.518 (0.064)	0.510 (0.061)	0.524 (0.066)	<0.001
Waist circumference, cm, mean (SD)	82.9 (10.4)	84.7 (10.4)	81.3 (10.1)	<0.001
Below threshold (Men ≥ 90/Women ≥ 85 cm)	121,345 (67.2%)	58,271 (69.3%)	63,074 (65.4%)	
Energy intake, kJ/day, mean (SD)	7824 (2547)	8620 (2722)	7156 (2178)	<0.001
Physical activity, MET-min/week, mean (SD)	1537 (1919)	1716 (2149)	1381 (1678)	<0.001
Physical activity level				<0.001
Very low [0, 300)	47,972 (27.0%)	23,996 (29.1%)	23,976 (25.3%)	
Low [300, 600)	25,076 (14.1%)	10,643 (12.9%)	14,433 (15.2%)	
Medium [600, 1200)	32,141 (18.1%)	12,791 (15.5%)	19,350 (20.4%)	
High [1200, 3000)	43,632 (24.6%)	18,573 (22.5%)	25,059 (26.4%)	
Very high ≥3000	28,590 (16.1%)	16,591 (20.1%)	11,999 (12.7%)	

***Notes:*** Data are presented as *n* (%) for categorical variables and mean (SD) for continuous variables. BMI categories use Chinese cut-points: <18.5, 18.5–23.9, 24.0–27.9, and ≥28.0 kg/m^2^. Waist circumference threshold: men ≥ 90 cm, women ≥ 85 cm. Physical activity (PA) levels are defined by MET-min/week: very low [0, 300), low [300, 600), medium [600, 1200), high [1200, 3000), and very high ≥ 3000.

**Table 2:** Distribution of participants with excess adiposity *N* (%).

Indicator	Overall	Women	Men	*p*
Participants, *n*	180,480	96,415	84,065	
BMI ≥ 28.0 and (high waist circumference or WHtR ≥ 0.50)	25,897 (14.3%)	14,475 (15.0%)	11,422 (13.6%)	<0.001
BMI < 28.0 and (high waist circumference and WHtR ≥ 0.50)	35,191 (19.5%)	20,030 (20.8%)	15,161 (18.0%)	<0.001
BMI ≥ 40.0	307 (0.2%)	189 (0.2%)	118 (0.1%)	0.004
Total participants with excess adiposity	61,395 (33.9%)	34,537 (35.8%)	26,598 (31.6%)	<0.001

***Notes:*** Data are presented as *n* (%). Excess adiposity was classified as: (1) BMI ≥ 28.0 kg/m^2^ and (WC ≥ 90 cm in men or ≥85 cm in women or WHtR ≥ 0.50); (2) BMI < 28.0 kg/m^2^ and (WC ≥ 90 cm in men or ≥85 cm in women and WHtR ≥ 0.50); or (3) BMI ≥ 40.0 kg/m^2^. Abbreviations: BMI, body mass index; WC, waist circumference; WHtR, waist-to-height ratio; M, men; F, women.

## Data Availability

Data from the China Nutrition and Health Surveillance (CNHS) 2015 are not publicly available due to data governance and participant confidentiality. Access may be granted upon reasonable request and with permission of the data custodians.
